# The Diagnosis and Treatment of Impalpable Testes at King Salman Armed Forces Hospital, Tabuk, Saudi Arabia

**DOI:** 10.7759/cureus.6659

**Published:** 2020-01-15

**Authors:** Mohammad S Mohammad Alnoaiji, Asmaa Ghmaird, Eid H Alshahrani, Fatima A Qaisy, Rana S Alotaibi, Basmah I Albalawi, Abeer M Asiri, Yazeed A Alshehri, Rofaida A Alenzi, Manal E Alatawi, Sumayah A Alzahrani, Tahani Nasser Alrashidi

**Affiliations:** 1 Paediatric Surgery, King Salman Armed Forces Hospitals, Tabuk, SAU; 2 Pediatrics, University of Tabuk, Tabuk, SAU; 3 Pediatric Surgery, University of Bisha, Bisha, SAU; 4 Pediatric Surgery, University of Tabuk, Tabuk, SAU; 5 Pediatric Surgery, King Khalid University, Abha, SAU

**Keywords:** cryptorchidism, impalpable testis, laparoscopy, orchidopexy, surgical exploration, time-to-treatment, ultrasonography

## Abstract

Background

Impalpable testes may be caused by atrophy, congenital dysgenesis/agenesis, or the presence of testes at unusual sites. Early intervention can improve patient outcomes. The recommended age for surgery ranges from 6 to 18 months.

Objective

To investigate the diagnosis, treatment, and outcomes of impalpable testes and sensitivity and specificity of ultrasonography to diagnose impalpable testes at King Salman Armed Forces Hospital (KSAFH), Tabuk, Saudi Arabia.

Methods

We conducted a retrospective study to review cases of impalpable testes admitted to KSAFH, Tabuk, Saudi Arabia from January 1, 2015 to May 20, 2019. Fifty patients diagnosed with impalpable testes were treated surgically in our center during the period. Patients’ data were tabulated, and statistical analysis was performed using Statistical Package for Social Sciences software (SPSS, version 22; IBM, Armonk, NY).

Results

We included 50 patients in our study, with a total number of 66 impalpable testes. The median age at diagnosis was 7 months, while the median age at surgery was 17.5 months. The median interval between diagnosis and surgery was 8.5 months, with 44% of cases undergoing surgery after the age of 1.5 years. Two-thirds of the cases were unilateral. The most common site was intracanalicular (57.6%) followed by intra-abdominal (34.8%). The testicular size was average in 36.4%, small in 42.4%, and atrophic in 21.2% of the evaluated cases.

Conclusions

The overall sensitivity of ultrasonography was 56.1% (it correctly detected the location of 37 out of 66 impalpable testes). The sensitivity of ultrasonography for the detection of intra-abdominal testes was 43.5%, while that of intracanalicular testes was 71.1%.

## Introduction

Testes that are not normally found at the base of the scrotum are termed undescended testes (UDT) or maldescended testes. The condition is also referred to as cryptorchidism [1]. It is one of the most common congenital anomalies and is more common among premature infants. It occurs in 1-4% of full-term and 1-45% of preterm male neonates [2]. The testes normally descend spontaneously in the first months after birth; however, around 1-2% of all boys still have UDT at 3 months after birth [1]. Because the clinical evaluation and management of these cases depend on the presence and location of the testes, the most useful and practical classification of UDT categorizes these into palpable and impalpable testes; about 80% of all UDT is palpable [3] 

Imaging procedures are needed to diagnose impalpable testes to prevent unnecessary surgery. However, ultrasound remains unreliable for these purposes, with an overall sensitivity of only 45% and specificity of 78% [4]. Laparoscopy is now considered the first choice for intra-abdominal exploration of the impalpable testes. It is sometimes recommended to start with a scrotal incision to explore for a scrotal nubbin or hernia sac and then proceed to laparoscopy only if the nubbin is not identified. Any surgical procedure for impalpable testes should begin by examining the patient under anesthesia using a lubricant on the fingertips to reevaluate for the presence of a testis when the patient is relaxed. This examination is especially important for obese patients [5].

We aimed to investigate the diagnosis, treatment, outcomes of impalpable testes and sensitivity of ultrasonography to diagnose impalpable testes at King Salman Armed Forces Hospital (KSAFH), Tabuk, Saudi Arabia.

## Materials and methods

This retrospective study reviewed the hospital files of children who were diagnosed with impalpable testes and operated at KSAFH from January 1, 2015 to May 30, 2019. We obtained ethical approval from the KSAFH Research Ethics Committee. We were cautious with the patient data, and measures were taken to ensure that the data would not be used for any purposes outside of this study. We gave each subject a unique identifier code in the datasheet software to protect the patient's personal data (e.g., name, contact info). We enrolled all children with UDT from birth until 13 years old who were admitted within the specified time frame and diagnosed with impalpable testes. The inclusion criteria were as follows: patients diagnosed with impalpable testes and who were younger than 13 years old at the time of the operation. We excluded any patient who was older than 13 years of age at the time of the operation and anyone with incomplete data. We extracted the patients’ data from their medical records. We collected the following data: a) age at diagnosis and surgery; b) duration between the diagnosis and surgery; c) the laterality of the UDT (right, left or bilateral); d) whether testes were palpable on clinical examination; e) ultrasonography findings; f) type of surgical technique; g) intraoperative location and findings of testes; h) postoperative size and placement of testes; i) condition of the testes after the follow-ups; j) associated comorbidities and anomalies. Depending on the age of the patient at the time of surgery, we divided the patients into two groups: 18 months of age or less (group 1) and older than 18 months (group 2). Patients’ data were tabulated, and statistical analysis was performed using Statistical Package for Social Sciences software (SPSS, version 22; IBM, Armonk, NY). The description of the studied categorical variables was achieved with frequencies and absolute numbers. All numerical variables were checked for normality by the Shapiro-Wilk test. Abnormally distributed data were summarized as median, interquartile range (IQR: expressed as 25th-75th percentiles), and range.

## Results

This study included 50 patients who underwent surgical management for impalpable testes. The number of impalpable testes was 66. The median age at diagnosis was seven months (IQR: 3-18); the median age at surgery was 17.5 months (IQR: 12-26), and the median time interval between diagnosis and surgery was 8.5 months (IQR: 6-12) (Table 1).

**Table 1:** Age at diagnosis and surgery and the duration between diagnosis and surgery in this cohort

	Median	Interquartile range	Range
Age at diagnosis (months)	7.0	3.0-18.0	0.0-72.0
Age at surgery (months)	17.5	12.0-26.0	0.0 - 108.0
Duration between diagnosis at pediatric surgery clinic and surgery (months)	8.5	6.0-12.0	0.0-72.0

**Figure 1:**
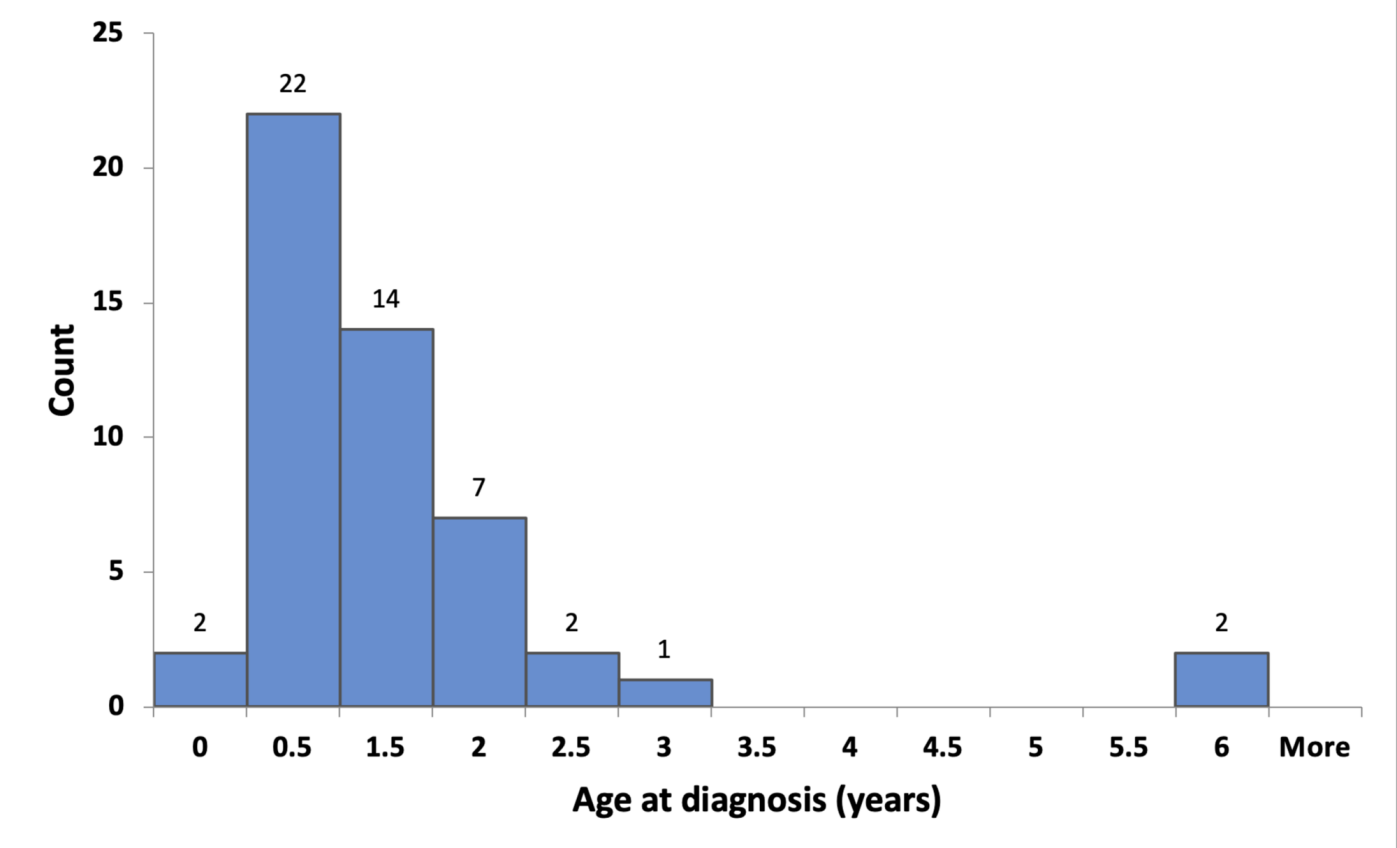
Age at diagnosis

Most patients (38 out of 50 patients, 76%) were diagnosed at or below the age of 1.5 years (Figure 1).

**Figure 2:**
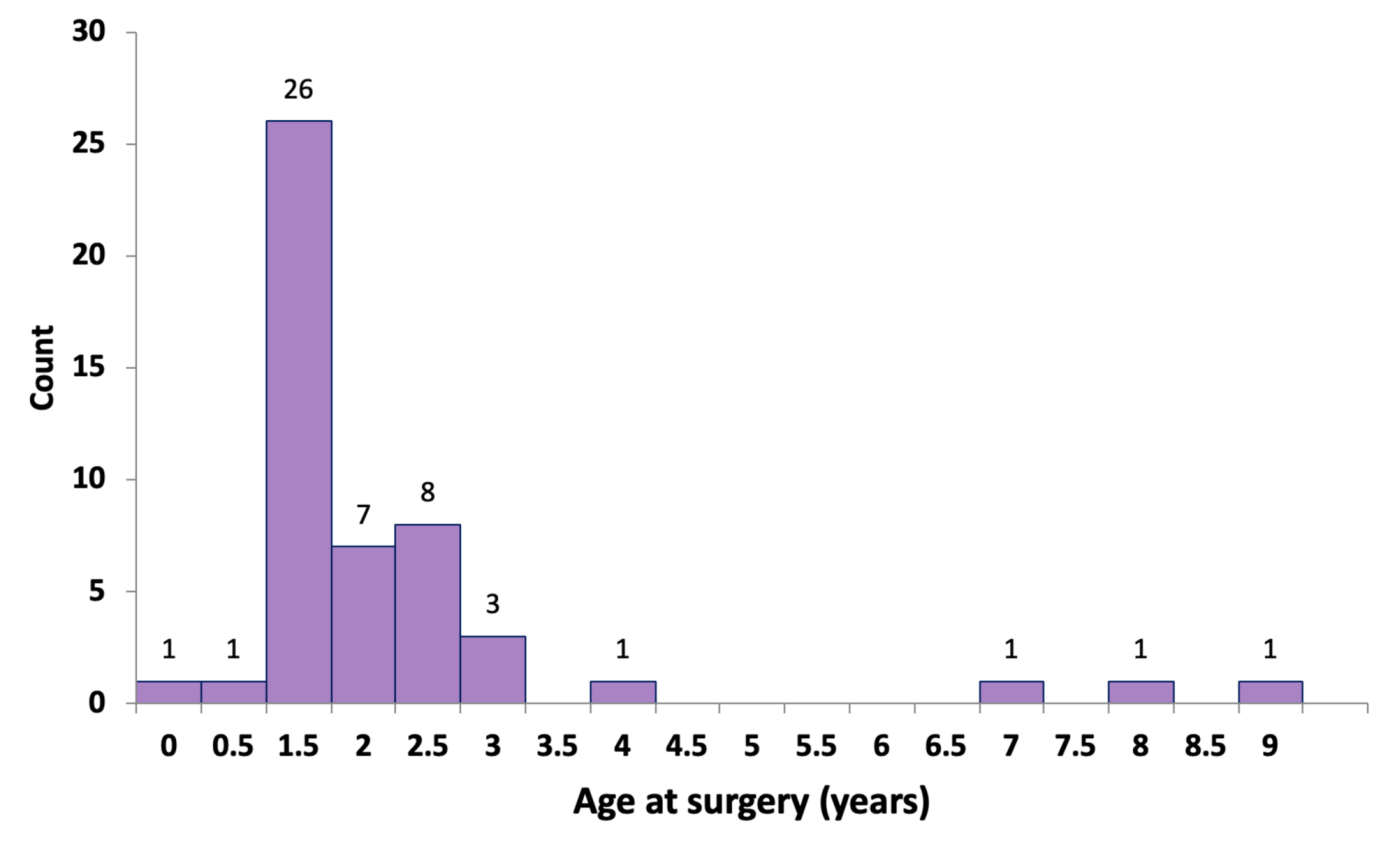
Age at surgery

However, 28 out of 50 patients (56%) underwent surgery at or before the age of 1.5 years (Figure 2).

**Figure 3:**
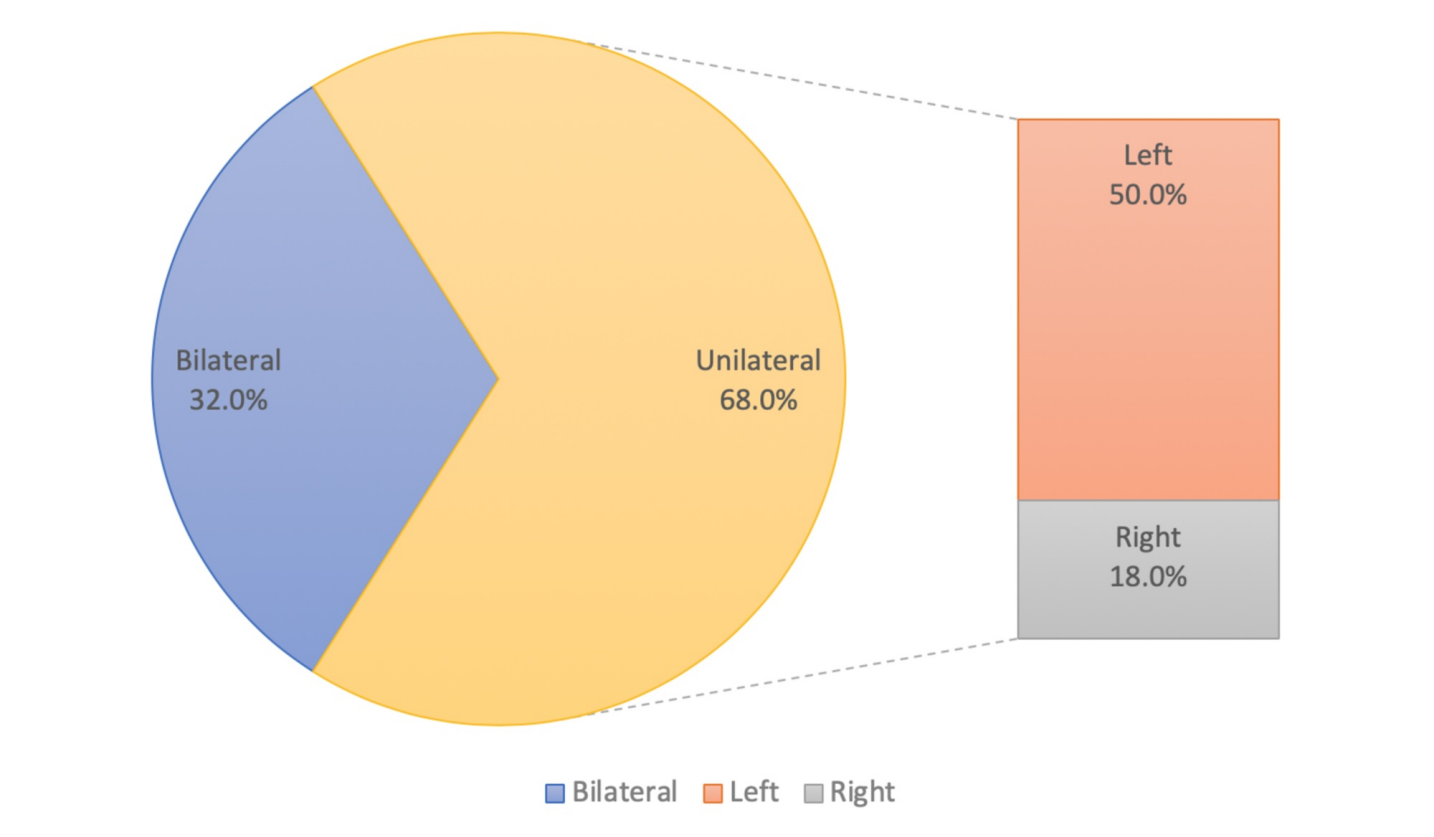
Site of impalpable testes in patients

About two-thirds of patients had unilateral impalpable testes (34 out of 50, 68%), while bilateral impalpable testes were encountered in about one-third of cases (16 out of 50, 32%). In unilateral cases, the left testes (25 out of 50, 50%) were affected more frequently than the right (nine out of 50, 18%) (Figure 3).

Examination with ultrasonography found that impalpable testes were intra-abdominal in 21.2% of cases (14 out of 66) and intracanalicular in 43.9% of cases (29 out of 66). The testes were not visualized in 34.8% of cases (23 out of 66). The surgical approach was open inguinal in 32 out of 66 cases (48.5%) and laparoscopic in 34 out of 66 cases (51.5%). During surgery, 38 out of 66 testes were intracanalicular (57.6%), 23 out of 66 were intra-abdominal (34.8%), three testes were ectopic (4.5%), and two testes were found in the inguinal region (3%). Only 24 out of 66 testes (36.4%) were average in size, while 28 out of 66 (42.4%) were small, and 14 out of 66 (21.2%) were atrophic (Table 2).

**Table 2:** Ultrasonographic and intraoperative findings N: number

		N	%
Ultrasonography findings	Intra-abdominal	14	21.2
	Intracanalicular	29	43.9
	Not visualized	23	34.8
Surgical management	Open	32	48.5
	Laparoscopic	34	51.5
	Stage I	26	39.4
	Stage II	8	12.1
Intraoperative location of testes	Intra-abdominal	23	34.8
	intracanalicular	38	57.6
	Inguinal	2	3.0
	Ectopic	3	4.5
Size of the testes during surgery	Average in size	24	36.4
	Small in size	28	42.4
	Atrophied testes	14	21.2

The sensitivity of ultrasonography for intra-abdominal testes was 43.5% (correctly detected the location of 10 out of 23 intra-abdominal testes) and 71.1% for intracanalicular testes (detected precisely 27 out of 38 intracanalicular testes). The overall sensitivity was 56.1% (correctly detected the location of 37 out of 66 impalpable testes) (Table 3).

**Table 3:** Comparison between the locations of impalpable testes as detected by ultrasonography and as found during the surgery

		Intraoperative location of testes			
		Ectopic	Inguinal	Intracanalicular	Intra-abdominal
Ultrasonography findings	Intra-abdominal	0 (0.0%)	0 (0.0%)	4 (10.4%)	10 (43.5%)
	Intracanalicular	0 (0.0%)	1 (50.0%)	27 (71.1%)	1 (4.3%)
	Not visualized	3 (100.0%)	1 (50.0%)	7 (18.4%)	12 (52.2%)

The size of the testes was average in 26 out of 66 cases (39.4%), small in 26 out of 66 (39.4%), and atrophic in 14 out of 66 (21.2%). Most of the testes maintained a normal position in the scrotum (40 out of 66, 60.6%), while the testes were in supra-scrotal position in seven out of 66 cases (10.6%) and high scrotal in five out of 66 cases (7.6%). A follow-up of the cases was completed except in one patient. After the follow-up period, there was an improvement in testicular growth: 29 out of 66 were normal in size (43.9%), while 21 out of 66 (31.8%) were small (Table 4).

**Table 4:** Postoperative findings and follow-ups N: number

		N	%
Postoperative size of the testes	Average	26	39.4
	Small	26	39.4
	Not applicable (atrophied testes)	14	21.2
Postoperative position of the testes	Normal position in scrotum	40	60.6
	Supra-scrotal	7	10.6
	High-scrotal	5	7.6
	Not applicable (atrophied testes)	14	21.2
Condition of the testes after follow-ups	Testes grew normally	29	43.9
	Testes small in size	21	31.8
	Follow-ups not finished	2	3.0
	Not applicable (atrophied testes)	14	21.2

Eighteen out of 50 patients (27.3%) had one or more associated co-morbidities. Eight out of 50 patients suffered from congenital heart disease (12.1%); five out of 50 patients (7.5%) had an inguinal hernia; two cases (3%) had a hydrocele; two cases had a subcoronal hypospadias (3%); one case presented with testicular torsion (1.5%). Other conditions were also present including asthma and insulin-dependent diabetes mellitus (two cases, 3%), deficiency of glucose-6-dehydrogenase, brain atrophy and hypotonia (two cases, 3%), adenoid hypertrophy (1 case, 1.5%), intrauterine growth retardation (one case, 1.5%), and stricture of external meatus (one case, 1.5%) (Table 5).

**Table 5:** Associated co-morbidities in the studied patients N: number

		N	%
Associated co-morbidities	Absent	48	72.7
	Present	18	27.3
	Congenital heart disease	8	12.1
	Inguinal hernia	5	7.5
	Hydrocele	2	3.0
	Sub-coronal hypospadias	2	3.0
	Testicular torsion	1	1.5
	Others	7	10.5

## Discussion

Infertility is one of the adverse outcomes that is desired to be decreased by performing early surgery for cryptorchidism [6]. Recent clinical guidelines recommend that treatment should be started at the age of 6 months and be finished by the age of 12 months or 18 months at the latest [7,8]. The median ages at diagnosis and surgery in our study were seven months and 17.5 months, respectively. Only 56% of the patients underwent surgery at or below the age of 18 months, which is the recommended age for orchidopexy. The median time interval between diagnosis and surgery was relatively long (8.5 months).

There have been very few studies on this topic in Saudi Arabia. Neel reviewed hospital charts of orchidopexies at two hospitals in Riyadh from 1998 to 2008 [9]. This study revealed that the patients who were diagnosed before the age of one year accounted for only 49.2% of the total cases, while the patients who were diagnosed after one year accounted for 45%, and 5.8% were diagnosed at pre-school checkups. Surprisingly, the study that the mean age at surgery was 54.8 months, and the boys who underwent the operation at age one were only 29.5% [9]. Sharif et al. did a retrospective review of all the operated cases of undescended testes from 2011 to 2013 at King Fahad Hospital, Al-Baha. Their results showed that the mean age at surgery was about three years. However, the boys who underwent orchidopexy above the age of two years represented 41.3% of the cases [10].

Alhazmi et al. reviewed the charts of patients who underwent orchidopexy in Riyadh from 2000 to 2010 and assessed referral time and waiting- list time. They concluded that the age at the time of surgery at their urology center was far from ideal (median: 46.7 months), primarily because of late referrals (median waiting list time: 15.2 months). They recommended a structured program to educate referring physicians [11]. Alsowayan et al. reviewed records of patients presenting to their center with undescended testes between 1996 and 2015 in Al Khobar. They found that the targeted recommended time frame was not usually met (median age at surgery was 25 months despite the international recommendation for orchidopexy being between the ages of 6-12 months). They assumed this to be related to late referral and the long waiting time for elective surgery (median age at diagnosis: 13.7 months; average waiting time for the operation: 4.8 months) [12].

Nah et al. conducted a retrospective review of all patients treated for undescended testes from 2007 to 2011 in Singapore. They found that despite early diagnosis in many patients with undescended testes, most were referred and operated after the age of one year (median age at referral: 1.1 years; median age at surgery: 1.6 years; median waiting time: 3.9 months). They recommended the establishment of community health initiatives that emphasize prompt referral to avoid delayed surgery and its associated adverse effects [13]. Hensel et al. analyzed orchidopexies between 2003 and 2012 in 13 German hospitals and found that 42% of patients who underwent orchidopexy were aged 4-17 years [14]. 

Schneuer et al. reviewed birth and hospital data of children from 2001 to 2011 in New South Wales, Australia. They reported that about 50% of patients were diagnosed within the first year after birth, and only 55% of all studied patients were treated within the recommended time frame. The median age at orchidopexy was found to be 16.6 months [15]. Wei et al. conducted an extensive study to determine the age at orchidopexy in China by reviewing orchidopexies performed in a large University Hospital between 1993 and 2014. They surveyed the general public's awareness of undescended testes as well as primary healthcare practitioners' current opinion on the desired age for performing orchidopexy and referral patterns. They found that the recommendations regarding the ideal age for orchidopexy were not adhered to (the median age at the surgery between 2010 and 2014 was 24 months) [16]. Williams et al. reviewed both the State Ambulatory Surgery Database in 2012 and the Pediatric Health Information System in 2015 in the US and found that about 70% of boys with UDT underwent orchidopexy at least six months later than the recommended age [17]. 

However, surgeries at earlier ages were reported by Alsaywid in Australia (median age at surgery: 11 months) and also by Bajaj and Upadhyay in New Zealand (median referral time; 2.95 months; median age at surgery: 12.63 months). Moreover, Bajaj and Upadhyay found that the surgery was done for 66% of the cases before the age of 18 months [18,19]. The majority of patients with impalpable testes can be diagnosed immediately after birth. The age at diagnosis depends mainly on the experience of the physician [20]. Poor knowledge and attitude of physicians about the management of impalpable testes and lack of awareness about clinical guidelines play an important role in delayed diagnosis and referral to pediatric surgery or pediatric urology. Shnorhavorian et al. conducted a study in the US, which demonstrated that 20% of the physicians did not refer children with undescended testes to surgery until puberty [21]. Moreover, a German survey showed that 38% of primary care pediatricians believed that orchidopexy should be performed after the first year of life [14]. In addition, Wei et al. found that 54% of healthcare practitioners believed that the optimum age for orchidopexy was between 24-36 months [16].

To prevent the complications of delayed intervention, pediatricians and family physicians should screen children in the early days after birth for congenital anomalies and refer the patients to specialists if issues are detected. In our study, about two-thirds of cases presented with unilateral impalpable testes (68%), and only one third (32%) had bilateral impalpable testes. Unilateral impalpable testes were more common on the left side than the right side (50% versus 18%). Other studies reported the right side to be more commonly affected [22-24]. Open surgical techniques and laparoscopy were performed in 48.5% and 51.5% of our patients, respectively. Sharif et al. conducted a retrospective study, which showed that the laparoscopic techniques were used only in 9.65% of the cases [1].

In the present study, the most common site of impalpable testes was found to be intracanalicular, followed by the intra-abdominal. These results were consistent with findings by Alsaywid and Mallikarjuna et al. [18,24]. However, this finding contradicted the result of Denes et al. [25]. The sensitivity of ultrasonography in our patients was 43.5% for the detection of intra-abdominal testes and 71.1% for the detection of intracanalicular testes, and the overall sensitivity was 56.1%. This finding is consistent with a previous study that showed the overall sensitivity of ultrasonography to be 45% [4]. However, our sample size was too small to accurately evaluate the sensitivity and specificity of ultrasonography in such patients.

Assessment of the size of impalpable testes during surgery revealed that 36.4% of testes were average; 42.4% were small, and 21.2% were atrophic. The rate of atrophic testes in the present study is higher than in previous studies (3.4% versus 11.8%) [1].

## Conclusions

In this study, the sensitivity of ultrasonography was found to be 43.5% for the detection of intra-abdominal testes and 71.1% for the detection of intracanalicular testes; the overall sensitivity was 56.1%. Laparoscopic exploration of impalpable testes has been found to be the most effective and accurate method to localize the condition.
